# The Black Hole Information Problem

**DOI:** 10.3390/e27060592

**Published:** 2025-05-31

**Authors:** Xavier Calmet, Roberto Casadio, Stephen D. H. Hsu

**Affiliations:** 1 Department of Physics and Astronomy, University of Sussex, Brighton BN1 9QH, UK; 2 Dipartimento di Fisica e Astronomia, Università di Bologna, via Irnerio 46, 40126 Bologna, Italy; casadio@bo.infn.it; 3 I.N.F.N., Sezione di Bologna, IS-FLAG, via B. Pichat 6/2, 40127 Bologna, Italy; 4 Alma Mater Research Center on Applied Mathematics-AM2, via Saragozza 8, 40123 Bologna, Italy; 5 Department of Physics and Astronomy, Michigan State University, East Lansing, MI 48823, USA; hsu.steve@gmail.com

This Special Issue of *Entropy* is focused on the black hole information paradox.

Nearly 50 years ago, Hawking argued that black holes cause pure states to evolve into mixed states [1,2]. Based on the causal properties of (classical) black hole spacetimes, he concluded that quantum information that falls into a black hole does not re-emerge in the form of radiation. Thus, the final radiation state left after a black hole has fully evaporated must be a mixed state, even if the hole was originally formed in a pure state.

Hawking’s argument has attracted significant attention, leading many theorists to describe the black hole information paradox as a fundamental clash between gravity and quantum mechanics.

Part of the difficulty in resolving this issue lies in the fact that we have a macroscopic object (a semi-classical black hole configuration), but we aim to characterize a very precise quantum aspect of its future state—whether that state is pure or mixed. In fact, we cannot perform this calculation even with a much more mundane system, such as a burning lump of coal. We simply accept on faith that the chemical reactions and resulting thermal fluctuations are unitary and preserve purity.

Recent work has significantly advanced our understanding of the black hole information paradox, exploring diverse approaches to reconcile quantum mechanics with gravity. For instance, the island formula and replica wormholes have emerged as key tools in computing semi-classical entropy, suggesting that information is preserved through quantum entanglement across spacetime regions [3,4]. Double-holographic models, leveraging the AdS/CFT correspondence, provide insights into evaporation dynamics by modeling black holes as dual to quantum systems [5]. Studies in two-dimensional dilaton gravity theories offer simplified frameworks to track information during evaporation, revealing unitary evolution in solvable models [6]. Significant work in the last few years [7,8,9,10,11,12] has focused on the fact that the late-time Hawking radiation state of a black hole is necessarily a macroscopic superposition state. Even in Hawking’s leading order approximation, in which the emission amplitudes are thermal, there are many macroscopically distinct patterns of radiation that emerge (e.g., with somewhat more or less energy emitted in a particular direction, at a particular time). If quantum evolution (i.e., according to the Schrödinger equation) is linear, these macroscopically different patterns must correspond to different components of a superposition state. Because of momentum conservation, the black hole recoil trajectory (and hence the spacetime geometry itself) is in one-to-one correspondence (modulo coarse graining) with these different radiation patterns. Hence, the description of the evaporation process in terms of a single Penrose diagram leaves out an essential physical aspect. This simple observation suggests significant revisions to some of the standard formulations of the information paradox.

Perhaps there is still much more to say about Hawking’s paradox!

In this Special Issue, we have collected papers that address black hole evaporation and the information paradox in novel ways.

In The Quantum Memory Matrix: A Unified Framework for the Black Hole Information Paradox [13], Neukart, Brasher and Marx present their Quantum Memory Matrix hypothesis, which is designed to address the longstanding Black Hole Information Paradox rooted in the apparent conflict between Quantum Mechanics and General Relativity.

In Modification of Premises for the Black Hole Information Paradox Caused by Topological Constraints in the Event Horizon Vicinity [14], Jacak argues that at the rim of the photon sphere of a black hole, the quantum statistics transition takes place in any multi-particle system of indistinguishable particles, which passes through this rim to the inside. Furthermore, he argues that the related local departure from the Pauli exclusion principle restriction causes a decay of the internal structure of collective fermionic systems, including the collapse of Fermi spheres in compressed matter. He discusses the possible implications for the black hole information paradox.

In Corrected Thermodynamics of Black Holes in f(R) Gravity with Electrodynamic Field and Cosmological Constant [15], Xu, Zhang, Yang, Yang and Lu discuss the thermodynamics of black holes and how physics beyond general relativity can affect thermodynamical quantities. Specifically, they look at f(R) gravity. Their study reveals that the corrected black hole thermodynamics can be locally stable for some models, and that corrected systems undergo a Hawking–Page phase transition.

In Beyond Additivity and Extensivity of Entropy for Black Hole and Cosmological Horizons [16], 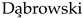 browski presents a comparative analysis of the plethora of nonextensive and/or nonadditive entropies that go beyond the standard Boltzmann–Gibbs formulation. After defining the basic notions of additivity, extensivity, and composability, he discusses the properties of these entropies and their mutual relations, if they exist. He argues that gravitational systems admit long-range interactions, which usually lead to a break of the standard additivity rule for thermodynamic systems composed of subsystems in Boltzmann–Gibbs thermodynamics.

In Lagrangian Partition Functions Subject to a Fixed Spatial Volume Constraint in the Lovelock Theory [17], Lu and Mann evaluate the quantum gravity partition function that counts the dimension of the Hilbert space of a simply connected spatial region of a fixed proper volume in the context of Lovelock gravity, generalizing the results for Einstein gravity. They find that there are sphere saddle metrics for a partition function at a fixed spatial volume in Lovelock theory. Those stationary points take exactly the same forms as in Einstein gravity. The logarithm of *Z* corresponding to a zero effective cosmological constant indicates that the Bekenstein–Hawking entropy of the boundary area and the one corresponding to a positive effective cosmological constant point to the Wald entropy of the boundary area. They also show the existence of zeroth-order phase transitions between different vacua, a phenomenon distinct from Einstein gravity.

In Tunneling between Multiple Histories as a Solution to the Information Loss Paradox [18], Chen, Sasaki, Yeom and Yoon discuss the information loss paradox associated with black hole Hawking evaporation. They revisit the evolution of the black hole entanglement entropy via the Euclidean path integral (EPI) of the quantum state and allow for the branching of semi-classical histories along the Lorentzian evolution. They posit that there exist at least two histories that contribute to EPI, where one is an information-losing history, while the other is an information-preserving one. At early times, the former dominates EPI; at the late times, the latter becomes dominant. By doing so, they recovered the essence of the Page curve and, thus, the unitarity, albeit with the turning point, i.e., the Page time, shifted toward the late time.

In Discreteness Unravels the Black Hole Information Puzzle: Insights from a Quantum Gravity Toy Model [19], Perez and Viollet argue that the black hole information puzzle can be resolved if two conditions are met. The first is that the information about what falls inside a black hole remains encoded in degrees of freedom that persist after the black hole completely evaporates. These degrees of freedom should be capable of purifying the information. The second is if these purifying degrees of freedom do not significantly contribute to the system’s energy, as the macroscopic mass of the initial black hole has been radiated away as Hawking radiation to infinity. They argue that the presence of microscopic degrees of freedom at the Planck scale provides a natural mechanism for achieving these two conditions without running into the problem of the large pair-creation probabilities of standard remnant scenarios. They demonstrate both key aspects of this mechanism using a solvable toy model of a quantum black hole inspired by loop quantum gravity.

## References

[1] Hawking S.W. (1976). Breakdown of predictability in gravitational collapse. Phys. Rev. D.

[2] Hawking S.W. (1976). Black Holes and Thermodynamics. Phys. Rev. D.

[3] Penington G. (2020). Entanglement Wedge Reconstruction and the Information Paradox. J. High Energy Phys..

[4] Almheiri A., Hartman T., Maldacena J., Shaghoulian E., Tajdini A. (2020). Replica Wormholes and the Entropy of Hawking Radiation. J. High Energy Phys..

[5] Almheiri A., Engelhardt N., Marolf D., Maxfield H. (2019). The Entropy of Bulk Quantum Fields and the Entanglement Wedge of an Evaporating Black Hole. J. High Energy Phys..

[6] Almheiri A., Mahajan R., Maldacena J., Zhao Y. (2020). The Page Curve of Hawking Radiation from Semiclassical Geometry. J. High Energy Phys..

[7] Calmet X., Casadio R., Hsu S.D.H., Kuipers F. (2022). Quantum Hair from Gravity. Phys. Rev. Lett..

[8] Calmet X., Hsu S.D.H. (2022). Quantum hair and black hole information. Phys. Lett. B.

[9] Calmet X., Hsu S.D.H. (2024). Replica Wormholes and Quantum Hair. arXiv.

[10] Calmet X., Hsu S.D.H., Sebastianutti M. (2023). Quantum gravitational corrections to particle creation by black holes. Phys. Lett. B.

[11] Calmet X., Hsu S.D.H. (2022). A brief history of Hawking’s information paradox. EPL.

[12] Calmet X., Hsu S.D.H. (2024). Black Hole Information, Replica Wormholes, and Macroscopic Entanglement. arXiv.

[13] Neukart F., Brasher R., Marx E. (2024). The Quantum Memory Matrix: A Unified Framework for the Black Hole Information Paradox. Entropy.

[14] Jacak J.E. (2024). Modification of Premises for the Black Hole Information Paradox Caused by Topological Constraints in the Event Horizon Vicinity. Entropy.

[15] Xu M., Zhang Y., Yang L., Yang S., Lu J. (2024). Corrected Thermodynamics of Black Holes in f(R) Gravity with Electrodynamic Field and Cosmological Constant. Entropy.

[16] Da̧browski M.P. (2024). Look Beyond Additivity and Extensivity of Entropy for Black Hole and Cosmological Horizons. Entropy.

[17] Lu M., Mann R.B. (2024). Lagrangian Partition Functions Subject to a Fixed Spatial Volume Constraint in the Lovelock Theory. Entropy.

[18] Chen P., Sasaki M., Yeom D.H., Yoon J. (2023). Tunneling between Multiple Histories as a Solution to the Information Loss Paradox. Entropy.

[19] Perez A., Viollet S. (2023). Discreteness Unravels the Black Hole Information Puzzle: Insights from a Quantum Gravity Toy Model. Entropy.

